# The Role of Pituitary Adenylate Cyclase-Activating Polypeptide (PACAP) Signaling in the Hippocampal Dentate Gyrus

**DOI:** 10.3389/fncel.2020.00111

**Published:** 2020-04-30

**Authors:** Gregory C. Johnson, Rodney Parsons, Victor May, Sayamwong E. Hammack

**Affiliations:** ^1^Department of Psychological Science, College of Arts and Sciences, University of Vermont, Burlington, VT, United States; ^2^Department of Neurological Sciences, Larner College of Medicine, University of Vermont, Burlington, VT, United States

**Keywords:** pituitary adenylate cyclase-activating polypeptide, PAC1 receptor, hippocampus, dentate gyrus, contextual fear conditioning

## Abstract

Pituitary adenylate cyclase-activating polypeptide (PACAP, *ADCYAP1*) dysregulation has been associated with multiple stress-related psychopathologies that may be related to altered hippocampal function. In coherence, PACAP- and PAC1 receptor (*ADCYAP1R1*)-null mice demonstrate changes in hippocampal-dependent behavioral responses, implicating the PACAPergic system function in this structure. Within the hippocampus, the dentate gyrus (DG) may play an important role in discerning the differences between similar contexts, and DG granule cells appear to both highly express PAC1 receptors and receive inputs from PACAP-expressing terminals. Here, we review the evidence from our laboratories and others that PACAP is an important regulator of activity within hippocampal circuits, particularly within the DG. These data are consistent with an increasing literature implicating PACAP circuits in stress-related pathologies such as post-traumatic stress disorder (PTSD) and implicate the hippocampus, and in particular the DG, as a critical site in which PACAP dysregulation can alter stress-related behaviors.

Pituitary adenylate cyclase-activating polypeptide (PACAP, *ADCYAP1*) dysregulation has been associated with multiple stress-related psychopathologies (Kormos and Gaszner, 2013; Hammack and May, 2015; Lutfy and Shankar, 2019) and we and others have reported that a single nucleotide polymorphism (SNP) in the PAC1 receptor gene (*ADCYAP1R1* rs2267735) as well as circulating PACAP levels were associated with post-traumatic stress disorder (PTSD; Ressler et al., 2011). These findings were consistent with a growing literature demonstrating central PACAP signaling as critical for stress responding and fear- and anxiety-like behavior in rodents. PTSD is characterized by several features, which may represent dysregulation in several distinct neural circuits. Notably, in the original report, Ressler et al. (2011) reported that the risk allele of rs2267735 was associated with hyper-arousal and dark-enhanced startles, which are behaviors that have been closely associated with activation of subregions of the bed nucleus of the stria terminals (BNST). These observations have corroborated several years of work in our laboratories demonstrating that BNST PACAP activation is necessary and sufficient for many of the behavioral sequelae produced by chronic stress (Hammack et al., 2010; Hammack and May, 2015), as well as cocaine self-administration (Miles et al., 2018, 2019). However, dysregulated fear extinction and fear discrimination are also hallmark features of PTSD (see Jovanovic et al., 2012; Sangha et al., 2020), and rs2267735 was also associated with compromised extinction and reduced discrimination between fear-conditioned stimuli (Ressler et al., 2011; Pohlack et al., 2015; Mercer et al., 2016; Ramikie and Ressler, 2016). These data suggest that PACAP dysregulation has effects in multiple neural circuits associated with PTSD symptoms, which may include regions of the amygdala, medial prefrontal cortex (mPFC) and hippocampus. There are several lines of evidence suggesting that PACAP activation has distinct and interesting actions in the central nucleus of the amygdala (CeA; Missig et al., 2017; Meloni et al., 2019; Varodayan et al., 2020), basolateral amygdala (BLA; Cho et al., 2012; Schmidt et al., 2015), and mPFC (Kirry et al., 2018, 2019), and some of this work has been reviewed elsewhere (Miles and Maren, 2019). Here, we discuss potential roles of PACAP regulating activity within the hippocampus, as well as the behavioral consequences of such regulation.

## Pituitary Adenylate Cyclase-Activating Polypeptide (PACAP)

PACAP is the archetypical member of the vasoactive intestinal peptide (VIP)-secretin-glucagon family of bioactive peptides and was isolated from ovine hypothalami based on its ability to stimulate adenylyl cyclase activity in anterior pituitary cells (Kimura et al., 1990; Miyata et al., 1990). Two α-amidated forms of PACAP arise from alternative posttranslational processing of the precursor molecule; PACAP38 has 38 amino acid residues [rat pro-PACAP(131-168)], while PACAP27 corresponds to the amino terminus of PACAP38 [proPACAP(131-157)]. Despite similarities in endoproteolytic processing by PC1 and PC2 (prohormone convertase 1 and 2, respectively) at dibasic amino acid processing sites, the levels of PACAP38 predominate in most tissues, although the ratio of the PACAP38: PACAP27 appears tissue-specific (Arimura et al., 1991). PACAP27 exhibits 68% amino acid identity with VIP (Kimura et al., 1990; Miyata et al., 1990); the 28-amino acid VIP peptide is also α-amidated but unlike PACAP, does not have alternatively processed forms. PACAP appears to represent the ancestral peptide and from gene duplications, VIP/PACAP and glucagon/GLP-1/GIP peptides appear to arise from different branches of the cladistic tree (Sherwood et al., 2000). PACAP peptides are well conserved among species and widely distributed among central and peripheral tissues to implicate their evolutionary importance in maintaining physiological homeostasis (Sherwood et al., 2000; Vaudry et al., 2009).

## PACAP Receptors: Expression and Signaling

PACAP can bind to three Class B heptahelical G protein-coupled receptors (GPCR). The PAC1 receptor is selective for the two PACAP isoforms (PACAP27/PACAP38); the VPAC1 and VPAC2 receptors exhibit similar high affinities for PACAP and VIP peptides (Harmar et al., 2012; Blechman and Levkowitz, 2013). Unlike the VPAC1 and VPAC2 GPCRs which are preferentially coupled to Gαs and adenylyl cyclase activity, the PAC1 receptors can be dually coupled to Gαs and Gαq/11 to engage adenylyl cyclase and phospholipase C activities, respectively. In addition to these classical plasma membranes delimited signaling mechanisms, the PAC1 receptors have also been shown to internalize and transduce long term endosomal signaling, especially β-arrestin-mediated ERK activation, to deliver second messengers to intracellular sites with high spatial and temporal resolution (Calebiro et al., 2010; Scita and Di Fiore, 2010; McMahon and Boucrot, 2011; Irannejad et al., 2013). From these studies, the PAC1 receptor can activate a multitude and integrated sequelae of downstream signaling events for cellular responses. Adding to the complexity, PAC1 receptors are unique among the Class B receptors in that multiple receptor variants depend on the absence or presence of two 84-bp Hip and Hop cassettes that encode inserts into the third cytoplasmic loop of the GPCR. Hence the PAC1 receptor can be Null with neither Hip nor Hop inserts, just Hip alone, just Hop or HipHop (Spengler et al., 1993; Harmar et al., 2012; Blechman and Levkowitz, 2013). Depending on the cell type, the different PAC1 receptor isoforms can be differentially coupled to the diverse downstream signaling cascades. From receptor isoform analyses, all regions of the mammalian central nervous system, including humans, preferentially express the PAC1Null and PAC1Hop receptor variants; only postganglionic sympathetic neurons appear unique in the expression of just the PAC1Hop receptor variant (Braas and May, 1999).

In our work related to stress- and pain-responding, only BNST and CeA infusions with PACAP altered anxiety- or pain-related behaviors (Hammack et al., 2010; Missig et al., 2014, 2017; Roman et al., 2014); parallel studies with VIP infusions had no apparent effects suggesting PAC1 receptor signaling in these behaviors. In accord, BNST PACAP and/or PAC1 receptor expression was upregulated by chronic stress or pain states (Hammack et al., 2009, 2010; Lezak et al., 2014; Missig et al., 2017) whereas VIP or VPAC1/VPAC2 transcript levels in these regions were not sensitive to these conditions. The PACAP effects could be blocked by the PAC1 receptor antagonist PACAP(6-38); moreover, many of the effects of PACAP infusion in these areas were mimicked by the specific PAC1 receptor agonist maxadilan (Missig et al., 2014, 2017; Roman et al., 2014) to demonstrate definitively roles for PAC1 receptor signaling in these responses.

PAC1 receptors are abundant and widely distributed in the CNS (Hashimoto et al., 1996; Jaworski and Proctor, 2000). Within the hippocampus, early *in situ* hybridization studies demonstrated high levels of PAC1 receptor transcripts in the granule cell layer (GCL) of the dentate gyrus (DG). Indeed, DG PAC1 receptor transcript expression can be striking compared to other brain regions (Hashimoto et al., 1996; Jaworski and Proctor, 2000). Complementary studies using PACAP-Cre mice (Bradford Lowell, McLean Hospital, Harvard University) demonstrated PACAP fiber projections from hippocampal hilar mossy cells to the DG inner molecular layer (IML), which implicated the formation of short PACAPergic synaptic circuits between PACAP-expressing hilar mossy cells and the proximal dendrites on PAC1 receptor-expressing DG granule cells (see below, Condro et al., 2016). PACAP may also bind to DG VPAC1 receptors; there is little VPAC2 receptor expression in this region (Vertongen et al., 1997). In sum, these observations implicated roles for PACAP/PAC1 receptor signaling in modulating hippocampal functions, including cognition, and learning and memory with potential consequences for contextually-mediated fear behaviors.

## The Hippocampus, Learning, and Memory

The functional anatomy of the hippocampus has been extensively reviewed (Schultz and Engelhardt, 2014). The axons from the entorhinal cortex (EC) layer 2 neurons project *via* the perforant pathway to the dendrites of granule cells in the outer molecular layers of the DG (Dolorfo and Amaral, 1998a,b). The mossy fibers of DG granule cells in turn project to the hippocampal CA3 region (Amaral et al., 2007; Blaabjerg and Zimmer, 2007), which then project to the CA1 region *via* Schaeffer Collaterals (Ishizuka et al., 1990). Pyramidal cells from CA1 then project back to layer 5 of the EC (Amaral and Witter, 1989). In the dorsal hippocampus, these circuits have been extensively studied for their role in the processing of contextual information. For example, several studies have determined distinct functional roles for the DG in contextual learning processes. Besnard et al. (2014) demonstrated enhanced DG activity following contextual conditioning as well as during the retrieval of contextual conditioning. Moreover, precise pharmacological inhibition of DG activity demonstrated the necessity of DG function in the expression of contextual fear at short time intervals (Hernández-Rabaza et al., 2008). Given that the overall activity of DG neurons is low under basal conditions, the DG may be preferentially activated in the acquisition and retrieval of contextual memories.

Using a transgenic mouse model where the expression of channelrhodopsin is under the control of the immediate-early gene c-fos promoter, Liu et al. (2012) reactivated the same population of DG neurons that were active at the time of contextual memory encoding. The reactivation of these DG neurons was able to enhance freezing behavior, presumably by stimulating the retrieval of the original contextual fear memory (Liu et al., 2012). Since this original report, many others have demonstrated that the reactivation of DG neuronal activity, hypothesized to be the encoding of context, is sufficient to retrieve contextual memories (Redondo et al., 2014; Ryan et al., 2015; Roy et al., 2016). Conversely, silencing DG neurons originally activated during memory encoding reduced freezing responses to a fearful context (Denny et al., 2014), suggesting that DG neuronal activity is necessary for retrieval. Hence, the population of DG neurons activated during the encoding of a contextual memory may represent the trace of that memory, also termed the memory engram (Ghandour et al., 2019). Bernier et al. (2017) further clarified the role of DG activity by demonstrating that DG inactivation significantly reduced contextual discrimination between similar contexts, while not affecting discrimination between very different contexts. As the DG may be critically involved in discerning the differences between similar contexts, altered hippocampal function may result in fear generalization across multiple contexts, which is a hallmark symptom of PTSD.

## PACAP and The Hippocampus

Several studies have demonstrated that PACAP systems regulate hippocampal activity. For example, the fEPSP response upon perforant pathway stimulation is enhanced in the presence of PACAP (Kondo et al., 1997), and in accord, Matsuyama et al. (2003) observed in both PACAP and PAC1 receptor knockout mice (Otto et al., 2001) a significant decrement in long term potentiation (LTP) following high-frequency stimulation of the perforant path. Otto et al. (2001) interpreted from receptor immunocytochemical data that the PAC1 receptors were presynaptic on DG mossy fibers and suggested an impairment of LTP at the mossy fiber/CA3 synapse following afferent stimulation in PAC1 receptor knockout mice. A few other studies have attempted to determine the role of PACAP in specific hippocampal subregions, especially the CA1. Hence, synaptic strength at the Schaeffer collateral synapse is sensitive to PACAP (Kondo et al., 1997; Roberto and Brunelli, 2000; Roberto et al., 2001; Ciranna and Cavallaro, 2003), which may be mediated by PACAP effects on AMPA currents (Costa et al., 2009; Toda and Huganir, 2015), NMDA currents (Macdonald et al., 2005), or other intrinsic channel mechanisms (Taylor et al., 2014). Behaviorally, PAC1 receptor knockout mice demonstrated decrements in several hippocampal-dependent tasks, such as foreground contextual fear conditioning (Sauvage et al., 2000), and background contextual fear conditioning (Otto et al., 2001). Moreover, PACAP infusions into CA1 enhanced the consolidation of contextual fear conditioning, and antagonism of the PAC1 receptor at CA1 impaired fear consolidation as well as subsequent fear extinction (Schmidt et al., 2015). Despite these studies, there is limited evidence of PACAP innervation of CA1 or CA3. Recent studies with PACAP-EGFP mice demonstrated sparse PACAPergic innervation of CA1 or CA3 (Condro et al., 2016), which appeared to complement the *in situ* hybridization data showing proportionately much less PAC1 receptor transcript expression in these areas than other regions of the hippocampus (Hashimoto et al., 1996; Jaworski and Proctor, 2000). Hence, whether the early observations on PACAP-mediated hippocampal function reflected VIP or PACAP signaling on VPAC receptors remained unclear. However, as a result of the availability of more recent tools and models, there has been a refocus of PACAP roles in the hippocampus.

## PACAP and The DG: Anatomy and Physiology

**Figure 1:**
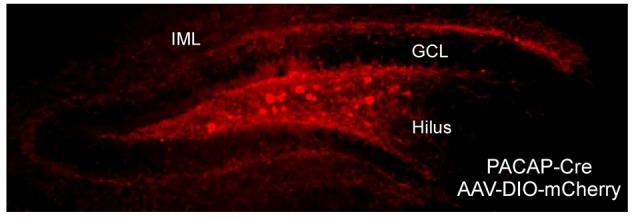
Neuronal pituitary adenylate cyclase-activating polypeptide (PACAP) expression and projection in the dentate gyrus (DG). The DG in PACAP-Cre mice was infused with Cre-dependent mCherry reporter. In coherence with work in PACAP-EGFP mice and previous PACAP and PAC1 receptor *in situ* hybridization studies, PACAP expression was in apparent hilar mossy cells which elaborated axonal projections predominantly to the inner molecular dendritic layer of granule cells. GCL, granule cell layer; IML, inner molecular layer.

In aggregate, the recent data from studies using the PACAP-EGFP and PACAP-Cre mice have been in alignment with previous PACAP and PAC1 receptor *in situ* hybridization studies, demonstrating that hippocampal PACAP expression is largely from hilar mossy cells which send projections predominantly to PAC1 receptor-expressing DG granule cell dendrites at the IML (Condro et al., 2016). From *in situ* hybridization data, PAC1 receptor mRNA expression is extensive in DG granule cells suggesting a role for PACAP in regulating DG activity. Previous *in situ* hybridization studies also identified PACAP-expressing neurons in DG hilar cells, and in coherence from PACAP-EGFP and PACAP-Cre mouse data, we and others have observed dense PACAP-positive axonal projections to the DG IML, where PACAP afferents likely synapse onto the dendrites of PAC1 receptor-expressing DG granule cells (Figure 1, Condro et al., 2016). Together, the anatomy suggests that a primary role of hippocampal PACAP signaling appears to be in the regulation of DG granule cell activity, and DG-related behaviors.

**Figure 2:**
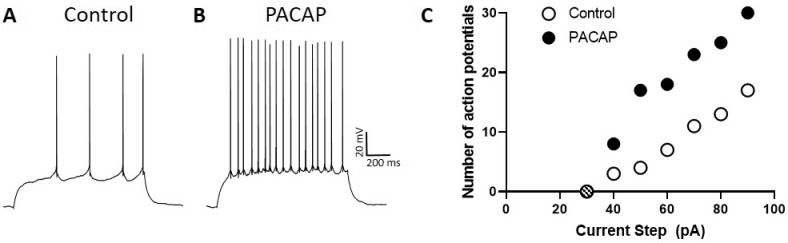
PACAP enhances the excitability of DG neurons. Trace **(A)**, representative patch-clamp recording in current-clamp mode from DG neuron demonstrating action potential generation elicited by a 1 s, 50 pA current step. Trace **(B)**, same DG neuron as in **(A)** demonstrating increased action potential generation under the same conditions but in the presence of PACAP. Dentate granule cell spiking upon PACAP infusion was greater than that elicited before peptide exposure. **(C)** Excitability curves produced by plotting the number of action potentials elicited by 1-s current steps of increasing strength. Note that the excitability curve for neurons treated with PACAP (filled circles) is steeper than that for untreated control cells (open circles). The hashed circle indicated no action potentials were elicited by a 30 pA step.

Using whole-cell recordings in current-clamp mode, Johnson et al. (2020) have observed that PACAP enhances the excitability of DG granule cells assessed by determining the number of action potentials elicited by 1-s depolarizing current steps of increasing intensity (Figure 2). Thus, PACAP increased the slope of the excitability curve, an effect of PACAP that was observed in other neuron types (Cho et al., 2012; Gupte et al., 2016; May and Parsons, 2017). Moreover, this increase in excitability was associated with a negative shift in the threshold for action potential generation, observed in nearly all DG granule cell neurons, due to post-synaptic actions of PACAP, and appeared to be independent of changes in input resistance and changes in resting membrane potential (Johnson et al., 2020). Importantly, while DG neurons demonstrate VPAC1 and low levels of VPAC2 receptor expression, the excitatory actions of PACAP were not mimicked by VIP, suggesting that the PACAP regulation of DG activity was PAC1 receptor-dependent. From these observations, we propose that PACAP release could serve to increase the excitability of DG granules cells that have been activated by other means, i.e., PACAP alone would likely not have independent neuronal effects without concurrent synergistic excitatory inputs. Hence the primary consequence of DG PAC1 receptor signaling would be to amplify the responses of activated neurons.

Following previous work, we investigated the signaling mechanisms and intrinsic membrane currents potentially contributing to the PACAP/PAC1 receptor enhancement of hippocampal neuronal excitability (Johnson et al., 2020). As noted above, there are many second messenger cascades downstream of PAC1 receptor activation, including plasma membrane delimited Gαs-mediated AC/cAMP and Gαq/11-mediated PLC/DAG/IP_3_ signaling. Also, both AC/cAMP and PLC/DAG/IP_3_ signaling, along with β-arrestin-mediated endosomal signaling following receptor internalization, can activate MEK/ERK signaling cascades.

In peripheral parasympathetic postganglionic cardiac neurons, all of these signaling pathways appeared to contribute to neuronal excitability by modulating different ionic conductances (Parsons et al., 2016; Tompkins et al., 2016; May and Parsons, 2017). Cyclic AMP generation was shown to gate the nonselective cationic H-current (Merriam et al., 2004; Tompkins et al., 2009), cAMP-activation of PKA was implicated in the activation of T-type calcium currents (Tompkins et al., 2015) and ERK signaling was shown to enhance a voltage-dependent sodium current possibly through phosphorylation of Nav1.7 (Tompkins et al., 2016). However, conditions that blunt clathrin-mediated receptor endocytosis, including treatment with the clathrin inhibitor Pitstop2 or the dynamin inhibitor dynasore, as well as decreasing ambient temperature, essentially eliminated the PACAP effect on cardiac neuron excitability (Merriam et al., 2013; May et al., 2014; Tompkins et al., 2018; Parsons and May, 2019). Thus receptor internalization and recruitment of endosomal signaling was a critical mechanism. While receptor internalization has been associated primarily with desensitization mechanisms, these views are being reconsidered; these PACAP/PAC1 receptors signaling studies were unique and among those implicating receptor endosomal signaling mechanisms as important drivers of neuronal excitability.

The signaling mechanisms underlying PACAP-enhanced DG granule cell excitability appeared different from those in peripheral cardiac neurons. Interestingly, from inhibitor studies, neither the AC/cAMP/PKA nor PLC/DAG/IP_3_ signaling appeared to contribute to PACAP modulation of DG cell excitability. In contrast, treatment with the MEK inhibitor, PD98059, virtually eliminated PACAP-enhanced induced excitability of DG granule cells (Johnson et al., 2020). Also, like cardiac neurons, the excitatory effects of PACAP on DG granule cells were essentially eliminated by treatment with the cell-permeable clathrin-mediated endocytosis inhibitor Pitstop2. Thus the results in aggregate suggested that for hippocampal DG cells, PAC1 receptor endosomal recruitment of MEK/ERK signaling represents the primary second messenger mechanism contributing to the PACAP modulation of neuronal excitability in DG granule cells (Johnson et al., 2020). These results appeared consistent with our other reports investigating PAC1 receptor signaling in other CNS pathways (Merriam et al., 2004; May et al., 2014; Missig et al., 2017; Miles et al., 2019). GPCR endosomal signaling, as opposed to membrane-bound activation of G-protein pathways, has been described to produced sustained long-lasting activation that is less sensitive to extracellular events that normally regulate signaling, such as transmitter diffusion and/or inactivation. Perhaps not surprisingly, the excitatory effects of PACAP in the *ex vivo* electrophysiological preparation were long-lasting even after peptide ligand washout. This mechanism for producing long-lasting changes in neuronal excitability may have important behavioral consequences, although the temporal dynamics of the behavioral effects of PACAP are largely unexplored. PACAP has been shown to alter the activity of several ion channels that regulate the excitability of neurons and ongoing studies focus on the identification of ionic currents in DG cells that potentially are the target of ERK phosphorylation.

## PACAP and The DG: Behavior

As noted earlier, DG granule cells play important roles in contextual fear conditioning, acquisition and retrieval, and given that PACAP may produce long-lasting changes in neuronal excitability of DG neurons (Johnson et al., 2020), PACAP may play a role in regulating these processes. As PACAP appears to augment DG neuronal activity to excitatory inputs, PACAP administration before training may facilitate contextual fear acquisition by enhancing the rate of conditioning or altering freezing behaviors during extinction testing on subsequent days. Alternatively, DG PACAP infusions before re-exposure to conditioning context may enhance fear expression thereby decreasing the rate of extinction. Whether DG PACAP signaling affects fear conditioning acquisition and/or extinction is under study but preliminary results suggest PACAP has a more pronounced effect on the latter. In this particular paradigm, DG PACAP/PAC1 receptor activation before extinction may lead to persistent neuronal activity in the neurons that make up the contextual fear memory engram to delay extinction processes.

As noted above, a polymorphism in the PAC1 receptor has been associated with PTSD symptoms (Ressler et al., 2011) and carriers of the risk allele exhibit differential hippocampal-dependent function in a contextual conditioning task compared to subjects with the normal allele (Pohlack et al., 2015). A hallmark feature of PTSD is the compromised ability to extinguish fear memories, which may be consistent with the DG PACAP behavioral effects described above. Not surprisingly, the processing of contextual information is highly associated with fear extinction, where the context may be used to disambiguate between the original fear memory and the extinction memory. Hence, should PACAP alter the processing of contextual information, the original fear memory may persist contributing to the disorder. Hippocampal dysfunction has also been associated with fear generalization, a feature of PTSD where fear responses are expressed in situations or environments unrelated to the acquired context (Dunsmoor and Paz, 2015). DG PACAP signaling may also participate in this process. For example, if chronic stress-induced DG PACAP neuroplasticity and altered DG function were to obfuscate engrams encoding fear memories with non-threatening or safe engram representations, then a consequence may be behavioral abnormalities that participate in PTSD. As an illustration, if behavioral engram representations for celebratory fireworks were obfuscated or supplanted, from stress-mediated PACAP neuroplasticity, by engrams encoding fear from combat barrages, then the changes in DG function may reinforce other maladapted stress circuits leading to psychopathologies. These data, combined with data implicating the role of stress-related PACAPergic dysregulation in other limbic regions in the regulation of emotional behavior, continue to implicate maladaptations to central PACAP/PAC1 receptor systems in the development and expression of fear and anxiety-related behavioral disorders.

## Discussion

Neural circuits in the hippocampus have been highly implicated in the processing of contextual information, including paradigms associated with emotional (fear) learning. In particular, processing in the hippocampal DG may be particularly critical for processing subtle differences between similar contexts. Anatomical data suggest that DG granule cells highly express PAC1 receptors and receive dense input from PACAP-expressing axon terminals in the IML. And, physiological data suggest that PACAP enhances the excitability of DG granule cells *via* PAC1 receptor endosomal signaling that activates MEK/ERK pathways to regulate the function of intrinsic membrane currents (Johnson et al., 2020). Consistent with this anatomy and physiology, PACAP infusions into the hippocampal DG appear to enhance the retention of a contextual fear memory. These data are consistent with previous reports implicating dysregulations in PACAP systems with PTSD symptoms and suggest that the hippocampal DG may be an important target for the effects of PACAP dysregulation. These data support an expanding literature that also implicates bed nucleus of the stria terminalis (BNST), central nucleus of the amygdala (CeA), hypothalamic subregions as other key structures where PACAP activation regulates emotional behavior. Together these data suggest that central PACAP activation is a key event that coordinates the activity of many circuits associated with the behavioral response to threatening stimuli; hence, PACAP systems may also play a key role when severe or chronic stress produces emotional pathology. These systems may be key targets for future therapeutic approaches to stress-related psychiatric disorders.

## Author Contributions

GJ performed some of the studies described in the manuscript. GJ, RP, VM, and SH contributed to some of the experimental designs, writing and editing of the manuscript.

## Conflict of Interest

The authors declare that the research was conducted in the absence of any commercial or financial relationships that could be construed as a potential conflict of interest.
